# 4,9,12,15-Tetra­oxa-3,5,8,10,14,16-hexa­aza­tetra­cyclo­[11.3.0.0^2,6^.0^7,11^]hexa­deca-1(16),2,5,7,10,13-hexaen-3-ium-3-olate monohydrate

**DOI:** 10.1107/S160053681200774X

**Published:** 2012-02-29

**Authors:** Yan-Shui Zhou, Bo-Zhou Wang, Kang-Zhen Xu

**Affiliations:** a Xi’an Modern Chemistry Research Institute, Xi’an 710065, Shaanxi, People’s Republic of China; b School of Chemical Engineering, Northwest University, Xi’an 710069, Shaanxi, People’s Republic of China

## Abstract

The organic mol­ecule in the title monohydrate, C_6_N_6_O_5_·H_2_O, presents an almost planar configuration, the greatest deviation from the least-squares plane through the atoms being 0.061 (1) Å for the O atom within the seven-membered ring. Each water H atom is bifurcated, one forming two O—H⋯N hydrogen bonds and the other forming O—H⋯N,O hydrogen bonds. The result of the hydrogen bonding is the formation of supra­molecular layers with a zigzag topology that stack along [001].

## Related literature

For background to related energetic materials, see: Sheremetev *et al.* (2010 ▶); Zhou *et al.* (2011 ▶); Rozhkov *et al.* (2004 ▶); Ovchinnikov *et al.* (2009 ▶).
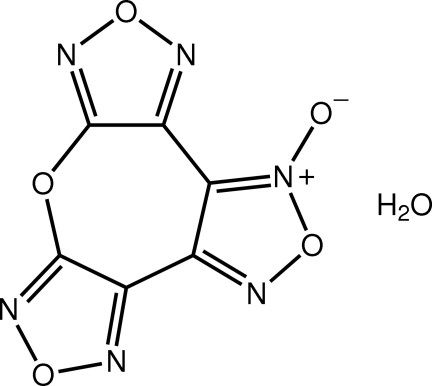


## Experimental

### 

#### Crystal data

C_6_N_6_O_5_·H_2_O
*M* *_r_* = 254.14
Monoclinic, 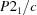

*a* = 9.324 (4) Å
*b* = 9.727 (4) Å
*c* = 10.391 (4) Å
β = 106.305 (6)°
*V* = 904.5 (6) Å^3^
*Z* = 4
Mo *K*α radiation
μ = 0.17 mm^−1^
*T* = 296 K
0.23 × 0.18 × 0.15 mm

#### Data collection

Bruker APEXII CCD diffractometer
Absorption correction: multi-scan (*SADABS*; Bruker, 2000 ▶) *T*_min_ = 0.962, *T*_max_ = 0.975
5058 measured reflections
2146 independent reflections
1808 reflections with *I* > 2σ(*I*)
*R*_int_ = 0.025

#### Refinement

*R*[*F* ^2^ > 2σ(*F* ^2^)] = 0.036
*wR*(*F* ^2^) = 0.097
*S* = 1.04
2146 reflections
172 parameters
All H-atom parameters refined
Δρ_max_ = 0.26 e Å^−3^
Δρ_min_ = −0.18 e Å^−3^

### 

Data collection: *APEX2* (Bruker, 2007 ▶); cell refinement: *SAINT* (Bruker, 2007 ▶); data reduction: *SAINT*; program(s) used to solve structure: *SHELXS97* (Sheldrick, 2008 ▶); program(s) used to refine structure: *SHELXL97* (Sheldrick, 2008 ▶); molecular graphics: *SHELXTL* (Sheldrick, 2008 ▶); software used to prepare material for publication: *SHELXTL*.

## Supplementary Material

Crystal structure: contains datablock(s) I, global. DOI: 10.1107/S160053681200774X/tk5030sup1.cif

Structure factors: contains datablock(s) I. DOI: 10.1107/S160053681200774X/tk5030Isup2.hkl

Supplementary material file. DOI: 10.1107/S160053681200774X/tk5030Isup3.cml

Additional supplementary materials:  crystallographic information; 3D view; checkCIF report

## Figures and Tables

**Table 1:** Hydrogen-bond geometry (Å, °)

*D*—H⋯*A*	*D*—H	H⋯*A*	*D*⋯*A*	*D*—H⋯*A*
O6—H1⋯N1^i^	0.78 (3)	2.52 (3)	3.068 (2)	128 (2)
O6—H1⋯N3^ii^	0.78 (3)	2.57 (3)	3.234 (2)	144 (3)
O6—H2⋯N5^iii^	0.90 (3)	2.40 (3)	3.201 (2)	149 (3)
O6—H2⋯O3^iii^	0.90 (3)	2.46 (3)	3.092 (2)	127 (3)

Symmetry codes: (i) 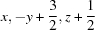; (ii) 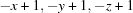; (iii) 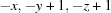.

**Fig. 1.**
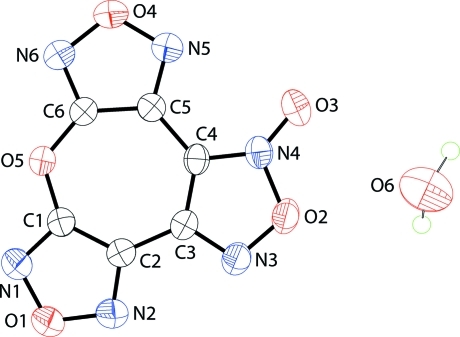
The molecular structure of (I), showing the atom-numbering scheme. Displacement ellipsoids are drawn at the 50% probability level and H atoms are drawn as spheres of arbitrary radius.

## supplementary crystallographic information

### Comment

Furazan-ether compounds are typical energetic materials known for their high energy-density, large heat of formation and low melting point (Sheremetev *et al.*, 2010; Zhou *et al.*, 2011; Rozhkov *et al.*, 2004; Ovchinnikov *et al.*, 2009). We used 3,4-dinitrofurzanfuroxan as a raw material for the synthesis of a new furazan-ether compound, bifurazano[3,4 - b:3',4'-f]furoxano[3'',4''-d]oxacyclohetpatriene, through a special etherifying reaction.

The organic molecule in the title monohydrate, C_6_N_6_O_5_.H_2_O, Fig. 1, presents an almost planar configuration with the greatest deviation from the least-squares plane through the atoms being 0.061 (1) Å for the O atom in the seven-membered ring.

### Experimental

At room temperature, 3,4-dinitrofurzanfuroxan (DNTF) (10.0 g, 0.0321 mol) and anhydrous sodium carbonate (4.6 g, 0.0434 mol) were taken into acetonitrile (25 mL). After reacting at 80 °C for 3.5 h, the resulting solution was transferred into water (80 mL), and then extracted with chloroform (60 mL) three times. The obtained organic phase was dried over anhydrous magnesium sulfate and then filtered. A white solid was obtained, yielding 3.8 g (50.1 %). *M*.pt. 265–267 K. ^13^C NMR (500 MHz, DMSO-d_6_): 160.50, 159.98, 144.39, 137.78, 135.52, 105.03 ppm. IR (KBr, cm^-1^): 1655, 1562, 1384, 1623, 1543, 1470, 997, 1151. Anal. Calcd for C_6_N_6_O_5_: C 30.51, N 35.59%. Found C 30.87, N 35.99%. Crystals were obtained by slow evaporation of its acetonitrile-water solution.

### Refinement

The water-H atoms were located from a difference map and freely refined.

### Crystal data

**Table:**

C_6_N_6_O_5_·H_2_O	*F*(000) = 512
*M**_r_* = 254.14	*D*_x_ = 1.866 Mg m^−^^3^
Monoclinic, *P*2_1_/*c*	Mo *K*α radiation, λ = 0.71073 Å
Hall symbol: -P 2ybc	Cell parameters from 3631 reflections
*a* = 9.324 (4) Å	θ = 2.9–28.1°
*b* = 9.727 (4) Å	µ = 0.17 mm^−^^1^
*c* = 10.391 (4) Å	*T* = 296 K
β = 106.305 (6)°	Block, yellow
*V* = 904.5 (6) Å^3^	0.23 × 0.18 × 0.15 mm
*Z* = 4	

### Data collection

**Table:**

Bruker APEXII CCD diffractometer	2146 independent reflections
Radiation source: fine-focus sealed tube	1808 reflections with *I* > 2σ(*I*)
Graphite monochromator	*R*_int_ = 0.025
φ and ω scans	θ_max_ = 28.2°, θ_min_ = 2.3°
Absorption correction: multi-scan (*SADABS*; Bruker, 2000)	*h* = −12→5
*T*_min_ = 0.962, *T*_max_ = 0.975	*k* = −12→12
5058 measured reflections	*l* = −13→13

### Refinement

**Table:**

Refinement on *F*^2^	Secondary atom site location: difference Fourier map
Least-squares matrix: full	Hydrogen site location: inferred from neighbouring sites
*R*[*F*^2^ > 2σ(*F*^2^)] = 0.036	All H-atom parameters refined
*wR*(*F*^2^) = 0.097	*w* = 1/[σ^2^(*F*_o_^2^) + (0.0389*P*)^2^ + 0.3369*P*] where *P* = (*F*_o_^2^ + 2*F*_c_^2^)/3
*S* = 1.04	(Δ/σ)_max_ < 0.001
2146 reflections	Δρ_max_ = 0.26 e Å^−^^3^
172 parameters	Δρ_min_ = −0.18 e Å^−^^3^
0 restraints	Extinction correction: *SHELXL97* (Sheldrick, 2008), Fc^*^=kFc[1+0.001xFc^2^λ^3^/sin(2θ)]^-1/4^
Primary atom site location: structure-invariant direct methods	Extinction coefficient: 0.009 (2)

### Special details

**Table:**

Geometry. All e.s.d.'s (except the e.s.d. in the dihedral angle between two l.s. planes) are estimated using the full covariance matrix. The cell e.s.d.'s are taken into account individually in the estimation of e.s.d.'s in distances, angles and torsion angles; correlations between e.s.d.'s in cell parameters are only used when they are defined by crystal symmetry. An approximate (isotropic) treatment of cell e.s.d.'s is used for estimating e.s.d.'s involving l.s. planes.
Refinement. Refinement of *F*^2^ against ALL reflections. The weighted *R*-factor *wR* and goodness of fit *S* are based on *F*^2^, conventional *R*-factors *R* are based on *F*, with *F* set to zero for negative *F*^2^. The threshold expression of *F*^2^ > σ(*F*^2^) is used only for calculating *R*-factors(gt) *etc*. and is not relevant to the choice of reflections for refinement. *R*-factors based on *F*^2^ are statistically about twice as large as those based on *F*, and *R*- factors based on ALL data will be even larger.

### Fractional atomic coordinates and isotropic or equivalent isotropic displacement parameters (Å^2^)

**Table:**

	*x*	*y*	*z*	*U*_iso_*/*U*_eq_	
C1	0.26986 (16)	1.03267 (15)	0.18452 (14)	0.0369 (3)	
C2	0.36461 (15)	0.95599 (14)	0.29024 (14)	0.0360 (3)	
C3	0.32627 (16)	0.87768 (14)	0.39420 (14)	0.0357 (3)	
C4	0.18293 (16)	0.86773 (13)	0.41014 (13)	0.0349 (3)	
C5	0.04614 (16)	0.92642 (14)	0.32715 (14)	0.0346 (3)	
C6	0.02700 (15)	1.00935 (14)	0.21087 (14)	0.0347 (3)	
N1	0.34793 (16)	1.08919 (16)	0.11423 (15)	0.0529 (4)	
N2	0.49995 (15)	0.96680 (16)	0.28215 (14)	0.0503 (3)	
N3	0.42478 (15)	0.81056 (14)	0.48677 (13)	0.0466 (3)	
N4	0.19345 (15)	0.79332 (13)	0.51789 (12)	0.0404 (3)	
N5	−0.08269 (15)	0.91226 (14)	0.34987 (14)	0.0456 (3)	
N6	−0.11096 (15)	1.04463 (14)	0.16482 (14)	0.0456 (3)	
O1	0.49242 (14)	1.04823 (15)	0.17392 (13)	0.0611 (4)	
O2	0.34869 (13)	0.75438 (12)	0.56793 (11)	0.0504 (3)	
O3	0.10766 (14)	0.75282 (12)	0.57909 (12)	0.0529 (3)	
O4	−0.18181 (12)	0.98452 (13)	0.25032 (12)	0.0519 (3)	
O5	0.12137 (11)	1.05420 (11)	0.14306 (10)	0.0425 (3)	
O6	0.21407 (18)	0.18370 (15)	0.41116 (15)	0.0614 (4)	
H1	0.290 (3)	0.221 (3)	0.442 (3)	0.096 (10)*	
H2	0.159 (4)	0.189 (4)	0.470 (3)	0.132 (12)*	

### Atomic displacement parameters (Å^2^)

**Table:**

	*U*^11^	*U*^22^	*U*^33^	*U*^12^	*U*^13^	*U*^23^
C1	0.0383 (7)	0.0365 (7)	0.0359 (7)	−0.0011 (5)	0.0104 (6)	0.0001 (5)
C2	0.0363 (7)	0.0351 (7)	0.0357 (7)	−0.0001 (5)	0.0086 (5)	−0.0038 (5)
C3	0.0391 (7)	0.0320 (7)	0.0334 (6)	0.0018 (5)	0.0059 (5)	−0.0015 (5)
C4	0.0431 (7)	0.0287 (6)	0.0315 (6)	−0.0004 (5)	0.0084 (5)	0.0004 (5)
C5	0.0370 (7)	0.0305 (6)	0.0363 (7)	−0.0017 (5)	0.0102 (5)	−0.0027 (5)
C6	0.0352 (7)	0.0328 (6)	0.0347 (6)	0.0009 (5)	0.0077 (5)	−0.0015 (5)
N1	0.0451 (8)	0.0623 (9)	0.0529 (8)	−0.0031 (6)	0.0162 (6)	0.0134 (7)
N2	0.0390 (7)	0.0614 (9)	0.0504 (8)	−0.0012 (6)	0.0123 (6)	0.0053 (6)
N3	0.0463 (7)	0.0464 (7)	0.0445 (7)	0.0033 (6)	0.0084 (6)	0.0070 (6)
N4	0.0485 (7)	0.0346 (6)	0.0381 (6)	−0.0018 (5)	0.0121 (5)	0.0014 (5)
N5	0.0402 (7)	0.0492 (7)	0.0486 (7)	−0.0001 (5)	0.0143 (6)	0.0035 (6)
N6	0.0388 (7)	0.0493 (7)	0.0474 (7)	0.0047 (5)	0.0100 (5)	0.0044 (6)
O1	0.0431 (6)	0.0820 (9)	0.0614 (8)	−0.0038 (6)	0.0197 (6)	0.0184 (7)
O2	0.0537 (7)	0.0486 (6)	0.0446 (6)	0.0047 (5)	0.0067 (5)	0.0137 (5)
O3	0.0644 (8)	0.0499 (7)	0.0495 (6)	−0.0045 (5)	0.0245 (6)	0.0093 (5)
O4	0.0357 (6)	0.0612 (7)	0.0592 (7)	0.0037 (5)	0.0141 (5)	0.0052 (6)
O5	0.0389 (5)	0.0492 (6)	0.0387 (5)	0.0043 (4)	0.0101 (4)	0.0117 (4)
O6	0.0607 (8)	0.0617 (8)	0.0641 (8)	−0.0195 (7)	0.0214 (7)	−0.0226 (6)

### Geometric parameters (Å, º)

**Table:**

C1—N1	1.290 (2)	C6—N6	1.2867 (19)
C1—O5	1.3458 (18)	C6—O5	1.3449 (17)
C1—C2	1.414 (2)	N1—O1	1.3744 (19)
C2—N2	1.292 (2)	N2—O1	1.3611 (19)
C2—C3	1.446 (2)	N3—O2	1.3598 (18)
C3—N3	1.3034 (19)	N4—O3	1.2186 (17)
C3—C4	1.395 (2)	N4—O2	1.4444 (18)
C4—N4	1.3135 (18)	N5—O4	1.3716 (18)
C4—C5	1.442 (2)	N6—O4	1.3773 (18)
C5—N5	1.295 (2)	O6—H1	0.78 (3)
C5—C6	1.422 (2)	O6—H2	0.90 (3)
			
N1—C1—O5	116.47 (13)	N6—C6—C5	109.95 (13)
N1—C1—C2	109.63 (14)	O5—C6—C5	133.21 (13)
O5—C1—C2	133.88 (13)	C1—N1—O1	104.97 (13)
N2—C2—C1	108.37 (13)	C2—N2—O1	106.04 (13)
N2—C2—C3	122.83 (13)	C3—N3—O2	106.07 (13)
C1—C2—C3	128.80 (13)	O3—N4—C4	136.06 (14)
N3—C3—C4	112.18 (13)	O3—N4—O2	117.75 (12)
N3—C3—C2	122.97 (14)	C4—N4—O2	106.19 (12)
C4—C3—C2	124.82 (12)	C5—N5—O4	105.73 (12)
N4—C4—C3	107.14 (12)	C6—N6—O4	104.91 (12)
N4—C4—C5	124.74 (13)	N2—O1—N1	110.99 (12)
C3—C4—C5	128.12 (13)	N3—O2—N4	108.41 (10)
N5—C5—C6	108.29 (13)	N5—O4—N6	111.13 (11)
N5—C5—C4	123.98 (13)	C6—O5—C1	123.18 (11)
C6—C5—C4	127.73 (13)	H1—O6—H2	108 (3)
N6—C6—O5	116.83 (13)		
			
N1—C1—C2—N2	−0.24 (18)	C3—C2—N2—O1	−179.44 (13)
O5—C1—C2—N2	177.82 (16)	C4—C3—N3—O2	−0.36 (16)
N1—C1—C2—C3	179.10 (14)	C2—C3—N3—O2	177.97 (12)
O5—C1—C2—C3	−2.8 (3)	C3—C4—N4—O3	179.40 (16)
N2—C2—C3—N3	−0.9 (2)	C5—C4—N4—O3	−0.4 (3)
C1—C2—C3—N3	179.87 (14)	C3—C4—N4—O2	−1.09 (14)
N2—C2—C3—C4	177.24 (14)	C5—C4—N4—O2	179.08 (12)
C1—C2—C3—C4	−2.0 (2)	C6—C5—N5—O4	0.01 (16)
N3—C3—C4—N4	0.97 (16)	C4—C5—N5—O4	179.09 (13)
C2—C3—C4—N4	−177.33 (13)	O5—C6—N6—O4	179.16 (12)
N3—C3—C4—C5	−179.21 (14)	C5—C6—N6—O4	−0.25 (16)
C2—C3—C4—C5	2.5 (2)	C2—N2—O1—N1	0.32 (19)
N4—C4—C5—N5	0.1 (2)	C1—N1—O1—N2	−0.46 (19)
C3—C4—C5—N5	−179.73 (14)	C3—N3—O2—N4	−0.33 (15)
N4—C4—C5—C6	178.95 (13)	O3—N4—O2—N3	−179.47 (12)
C3—C4—C5—C6	−0.8 (2)	C4—N4—O2—N3	0.92 (15)
N5—C5—C6—N6	0.16 (17)	C5—N5—O4—N6	−0.17 (16)
C4—C5—C6—N6	−178.88 (13)	C6—N6—O4—N5	0.26 (17)
N5—C5—C6—O5	−179.12 (15)	N6—C6—O5—C1	175.58 (13)
C4—C5—C6—O5	1.8 (3)	C5—C6—O5—C1	−5.2 (2)
O5—C1—N1—O1	−178.03 (13)	N1—C1—O5—C6	−175.61 (14)
C2—C1—N1—O1	0.41 (18)	C2—C1—O5—C6	6.4 (2)
C1—C2—N2—O1	−0.06 (17)		

### Hydrogen-bond geometry (Å, º)

**Table:**

*D*—H···*A*	*D*—H	H···*A*	*D*···*A*	*D*—H···*A*
O6—H1···N1^i^	0.78 (3)	2.52 (3)	3.068 (2)	128 (2)
O6—H1···N3^ii^	0.78 (3)	2.57 (3)	3.234 (2)	144 (3)
O6—H2···N5^iii^	0.90 (3)	2.40 (3)	3.201 (2)	149 (3)
O6—H2···O3^iii^	0.90 (3)	2.46 (3)	3.092 (2)	127 (3)

Symmetry codes: (i) *x*, −*y*+3/2, *z*+1/2; (ii) −*x*+1, −*y*+1, −*z*+1; (iii) −*x*, −*y*+1, −*z*+1.
